# Predation can shape the cascade interplay between heterothermy, exploration and maintenance metabolism under high food availability

**DOI:** 10.1002/ece3.11579

**Published:** 2024-06-25

**Authors:** Jan S. Boratyński, Karolina Iwińska, Martyna Wirowska, Zbigniew Borowski, Karol Zub

**Affiliations:** ^1^ Mammal Research Institute Polish Academy of Sciences Białowieża Poland; ^2^ University of Białystok Doctoral School in Exact and Natural Sciences Białystok Poland; ^3^ Department of Systematic Zoology Adam Mickiewicz University Poznań Poland; ^4^ Department of Forest Ecology Forest Research Institute Sękocin Stary Poland

**Keywords:** behavior, food availability, metabolic rate, predation, torpor

## Abstract

Maintenance metabolism as the minimum energy expenditure needed to maintain homeothermy (a high and stable body temperature, *T*_b_), reflects the magnitude of metabolic machinery and the associated costs of self‐maintenance in endotherms (organisms able to produce heat endogenously). Therefore, it can interact with most, if not all, organismal functions, including the behavior–fitness linkage. Many endothermic animals can avoid the costs of maintaining homeothermy and temporally reduce *T*_b_ and metabolism by entering heterothermic states like torpor, the most effective energy‐saving strategy. Variations in BMR, behavior, and torpor use are considered to be shaped by food resources, but those conclusions are based on research studying these traits in isolation. We tested the effect of ecological contexts (food availability and predation risk) on the interplay between the maintenance costs of homeothermy, heterothermy, and exploration in a wild mammal—the yellow‐necked mouse. We measured maintenance metabolism as basal metabolic rate (BMR) using respirometry, distance moved (exploration) in the open‐field test, and variation in *T*_b_ (heterothermy) during short‐term fasting in animals captured at different locations of known natural food availability and predator presence, and with or without supplementary food resources. We found that in winter, heterothermy and exploration (but not BMR) negatively correlated with natural food availability (determined in autumn). Supplementary feeding increased mouse density, predation risk and finally had a positive effect on heterothermy (but not on BMR or exploration). The path analysis testing plausible causal relationships between the studied traits indicated that elevated predation risk increased heterothermy, which in turn negatively affected exploration, which positively correlated with BMR. Our study indicates that adaptive heterothermy is a compensation strategy for balancing the energy budget in endothermic animals experiencing low natural food availability. This study also suggests that under environmental challenges like increased predation risk, the use of an effective energy‐saving strategy predicts behavioral expression better than self‐maintenance costs under homeothermy.

## Graphical abstract

Maintenance metabolism under homeothermy, costly behavior, and energy‐saving strategies (heterothermy as torpor use) are considered to be shaped by food resources, but those conclusions came from studies considering these traits in isolation. We aimed to test the effects of food availability (and predatory pressure) on the interplay between maintenance costs of homeothermy, torpor use, and exploration in a wild mammal. We found that the heterothermy as a compensation strategy is more important than the maintenance costs of homeothermy in the context of the behavioral expression under environmental challenges.

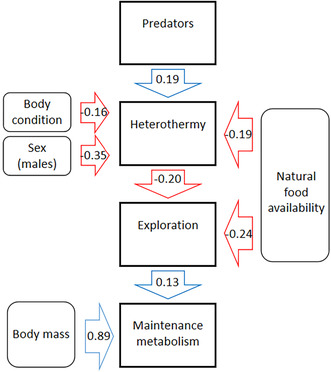

## 1 INTRODUCTION

Energy metabolism is considered to play a key role in survival in the context of ongoing global environmental changes—which are expected to disturb the predictability and availability of food resources (O'Connor et al., 2009). This is because the interaction between food supply and animal energetics constrains most, if not all, aspects of animal life (Auer et al., 2020). According to the ‘increased intake’ hypothesis, a more developed metabolic machinery allows an animal to more effectively transform and use available sources, with the extra energy being invested to improve fitness (Bennett & Ruben, 1979; Hayes et al., 1992; Hayes & Garland, 1995; Nilsson, 2002). However, a more developed metabolic machinery also entails higher costs spent on self‐maintenance, and finite energy, when allocated into one function, can limit other processes (Stearns, 1992). Hence, the ‘allocation’ hypothesis considers metabolic machinery costly and expects negative fitness‐related consequences (Careau et al., 2014; Gadgil & Bossert, 1970; Larivee et al., 2010; Steyermark, 2002). In endothermic animals characterized by the ability to produce heat as a byproduct of energy metabolism, the self‐maintenance costs of metabolic machinery correlate with the basal metabolic rate (BMR)—the minimum metabolism required for a resting animal to maintain a stable and high body temperature (*T*_b_) under thermoneutral conditions (McNab, 1997; Scholander et al., 1950).

Several hypotheses have aimed to explain how environmental conditions shape variation in BMR, the most comprehensive of which is the ‘food‐habits’ hypothesis. This hypothesis predicts that animals facing lower food quality, availability, or predictability should evolve (as a result of selection) or achieve during their development (as a result of plasticity) low BMR (Bozinovic et al., 2007; Cruz‐Neto & Jones, 2005; McNab, 2005). This links to the ‘context‐dependent’ hypothesis, which predicts that the association between fitness and metabolism varies with resource availability, i.e., high BMR can be beneficial when resources are available but disadvantageous when they are limited (Burton et al., 2011). Several interspecific studies have found support for the ‘food‐habits’ hypothesis (McNab, 1969, 1988, 2003; Muñoz‐Garcia & Williams, 2005; see also: Sabat et al., 2010). However, intraspecific studies have reported rather mixed results when testing its predictions (review in Cruz‐Neto & Bozinovic, 2004). This suggests that alternative mechanisms may explain resources and animal energetics linkage in natural systems, including the use of an effective energy‐saving strategy (Vuarin & Henry, 2014) that allows for avoiding the costs associated with self‐maintenance under homeothermy (Cruz‐Neto & Bozinovic, 2004).

The most effective strategy for saving energy in small endothermic animals is adaptive heterothermy, e.g. torpor—a state of inactivity characterized by reduced metabolic rate (MR) and *T*_b_ (Geiser, 2004: Heldmaier et al., 2004). A study of a variety of species indicated that hibernation (multi‐day torpor) and daily torpor (lasting <24 h) lower the energy expenditure associated with BMR by ~95% and ~65%, respectively (Ruf & Geiser, 2015). Several factors can trigger heterothermy use (Geiser & Brigham, 2012) but, the general function of torpor is to balance the energy budget, and individuals flexibly use this strategy in response to current energetic requirements (Boratyński et al., 2018; Boyles et al., 2007; Bozinovic et al., 2007; Eto et al., 2015; Fjelldal et al., 2021; Vuarin et al., 2013; Wojciechowski et al., 2007). Moreover, studies have found consistent among‐individual variation in torpor use, and less or more heterothermic animals co‐occur within populations (Boratyński et al., 2019; Dammhahn et al., 2017; Nespolo et al., 2010; Tapper et al., 2021), suggesting the existence of a continuum in thermoregulatory strategies (Angilletta et al., 2010; Boratyński et al., 2019; Boyles et al., 2013). Several studies have been carried out in laboratory conditions to test the effects of food shortage on animal thermoregulation at the intraindividual level (Vuarin & Henry, 2014). However, whether the consistent among‐individual variation in heterothermy is shaped by food resources in the natural environment is not well explored.

Food abundance is considered to be the main driver of changes in rodent population density as a result of its effects on the fitness of individuals (Boutin, 1990; Desy et al., 1990; Haapakoski et al., 2012; Taitt, 1981; Taitt & Krebs, 1981). The mechanism is likely mediated by an interaction between animal behavior and physiology, and its effects on individual reproduction and survival. The ‘performance’ model rooted in the ‘increased‐intake’ hypothesis considers energy transformation by metabolic machinery and thus, high BMR as crucial for the expression of costly behaviors (Careau et al., 2008). However, the ‘compensation’ model predicts that the high maintenance costs of metabolic machinery (the high BMR) may result in energy allocation trade‐offs when distinct functions have to be supported. Accordingly, high BMR should correlate negatively with behavioral expression (Careau et al., 2008), especially under low resource availability and/or other environmental disturbances (Iwińska et al., 2024). Therefore, the physiology–behavior linkage should be context‐dependent, and be potentially modified by environmental factors, as well as intrinsic interindividual differences. Energy‐saving strategies, such as heterothermy, can alter the predictions derived from these models. Intuitively, heterothermy should not only modify the linkage between BMR and behavior (by changing the context of energy management), and associated energy savings can be an even more significant driver in shaping animal behavior than the costs or limitations of metabolic machinery under homeothermy.

Here we aimed to examine the influence of natural and supplementary food resources on the potential interplay between self‐maintenance costs of homeothermy, heterothermy use (daily torpor), and exploratory behavior in wild‐caught yellow‐necked mice *Apodemus flavicollis*. In this small food‐hoarding rodent (Vander Wall, 1990), population dynamics are shaped by temporally variable and unpredictable changes in food availability—such as synchronous mass seed production (masting) of deciduous tree species (Bogdziewicz et al., 2016; Czeszczewik et al., 2020; Pucek et al., 1993; Stenseth et al., 2002; Zwolak et al., 2016). In our previous study, we found that heterothermy and BMR are lower in mice during masting than in non‐masting years (Boratyński et al., 2019, 2021). Similarly, in eastern chipmunks *Tamias striatus* maintenance metabolism and heterothermy use were found to be higher during non‐masting than in masting years (Careau, Réale, et al., 2013; Dammhahn et al., 2017). Thus, we expected that supplementary and natural food abundance would negatively correlate with both of these traits. In non‐masting years, yellow‐necked mice maintain larger home ranges than in masting years (Stradiotto et al., 2009); other studies have found that supplementary feeding generally results in reduced individual home ranges in many species (review in Boutin, 1990). Explorative individuals travel more and occupy larger home ranges and/or core areas (Aliperti et al., 2021; Boon et al., 2008; Montiglio et al., 2012; Schirmer et al., 2019; Wauters et al., 2021). Thus, we expected that in yellow‐necked mice explorative behavior would correlate negatively with natural and supplementary food abundance. Finally, we tested plausible causal links between the studied traits (using a path analysis), expecting that behavioral expression (1) is primarily limited by metabolic machinery, (2) is primarily shaped by the use of the energy‐saving mechanism, or (3) itself affects these two physiological functions.

## 2 MATERIALS AND METHODS

### 2.1 Study site and animals

**FIGURE 1:**
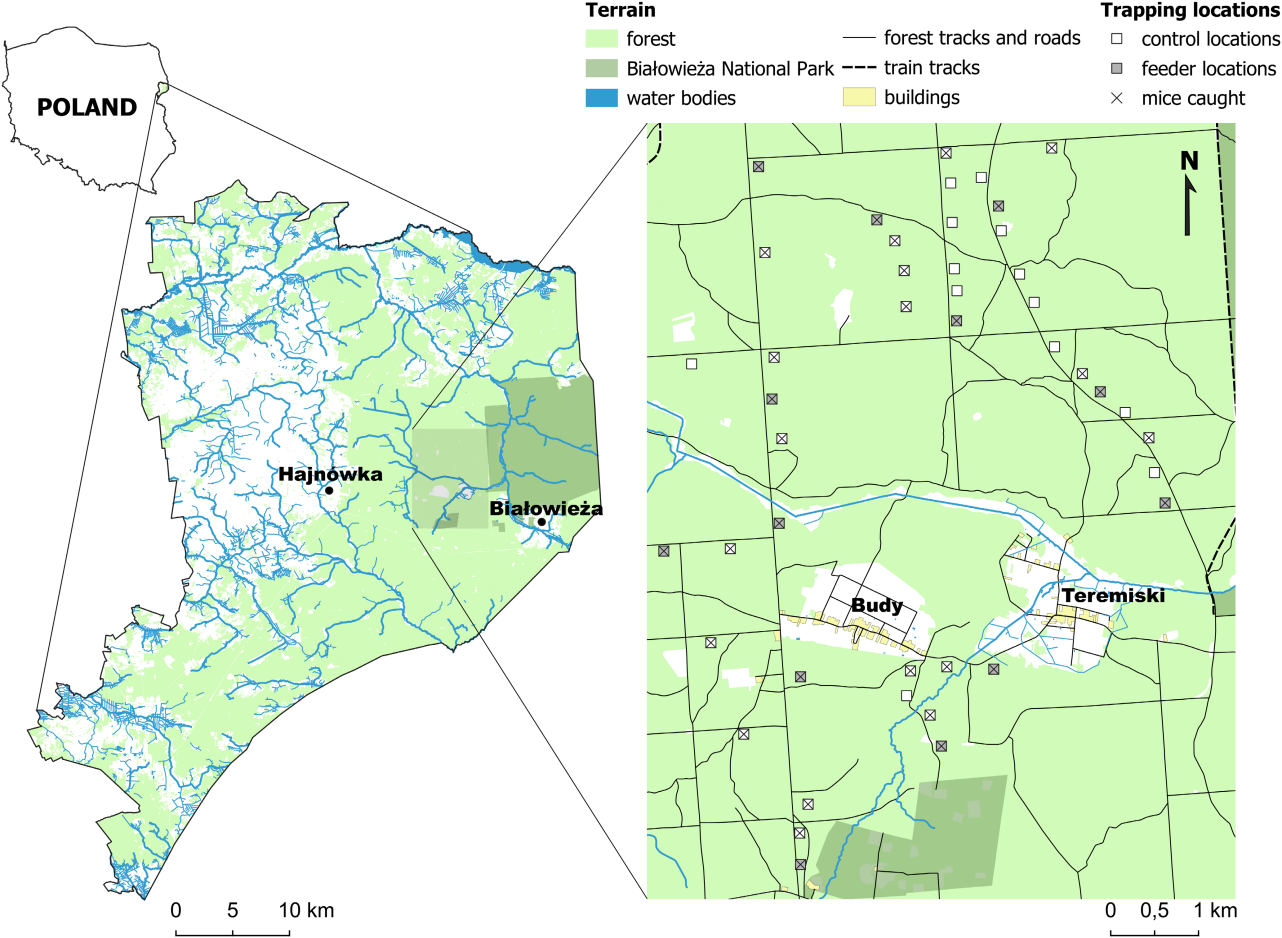
Map of the study area, presenting locations of trapping points and feeders.

The study was performed between September 2021 and March 2022 in the Polish part of the Białowieża Forest (Eastern Poland). We evaluated natural seed availability in autumn (see details below) and captured animals in winter at 44 locations (at least ~400 m apart: Figure 1). We chose these locations based on the occurrence of at least one old pedunculate oak *Quercus robur*, as its seeds are the main food resource affecting the winter survival of mice in Białowieża Forest (Czeszczewik et al., 2020). Another tree species significant for mouse population dynamics is hornbeam *Carpinus betulus*, widespread across the study site (Pucek et al., 1993). At each location, we placed one wooden box (65 × 40 × 22 cm) with openings that allowed access for mice but not their predators. Moreover, 13 of these wooden boxes (randomly selected to be at least ~1000 m apart) were kept full of hazelnuts and sunflower seeds between autumn, and the trappings in winter. All feeders were checked weekly and refilled with seeds when needed. At each location, natural seed availability was evaluated during three sessions: early autumn (7–9 September), middle autumn (21–23 September), and late autumn (13–16 October). During each session, 50 × 50 cm wooden frames were placed on the ground, and the seeds inside them were collected. We placed frames in two ways: (1) a frame was placed under the crown of each oak (1–2 m from the trunk) present within a 30 m radius of each wooden box; (2) additional five frames were placed at each location (next to the wooden box, and 10 m to its North, East, West, and South). In the first approach we collected acorns, and during the second, we collected other tree seeds, such as hornbeam nuts. Collected seeds were dried at 105°C for 18 h (International Seed Testing Association, 1985) and then weighed to the nearest 0.01 g (SBS‐LW‐2000A, Steinberg Systems, Berlin, Germany). Between the end of January and the beginning of March, mice were captured using seven wooden traps placed in each wooden box for two consecutive days (traps were inspected twice a day: before sunset and after sunrise). Before trapping (between 30th December and ~15th January), we monitored the activity of predators at each location for subsequent 5 days using a camera trap (model: 119740, Bushnell, Kowloon, Hong Kong, and China). Camera traps (motion activated) were placed at a height of ~1 m and ~5 m away from the wooden‐boxes and were set to record 15 s videos at 1 min intervals.

In the early morning (no mice were captured during daytime), animals successfully captured (*N* = 117) at 31 locations (Figure 1) were transported to a laboratory in the Mammal Research Institute in Białowieża. On the same day before midday, each animal was tagged with a thermosensitive programmable identification transponder (model: IPTT‐300, Biomedic Data Systems Inc., Seaford, US). Transponders were precalibrated in a water bath at five temperatures (range = 18–42°C) against a precise (0.1°C) mercury‐in‐glass thermometer (Jenatherm N, Germany). Implantation was done under a 2% mixture of isoflurane (Iso‐Vet) and oxygen anesthetic. While mice were still anesthetized, we measured their head width (HW) with calipers (0.1 mm precision; Measy DG, Ecotone, Gdańsk, Poland) and weighed them to the nearest 0.1 g (ScoutPro 200; Ohaus, Parsippany, NJ, USA). Between measurements in a laboratory, animals were kept in standard individual cages (model: 1264, Tecniplast, Buguggiate, Italy) at 16 °C, under natural photoperiod, with access to rodent food (Megan, Kraków, Poland), apples, and water *ad libitum*.

### 2.2 Laboratory measurements

After a ~week (5–9 days) of habituation to laboratory conditions, we quantified the behavior of each mouse during an open‐field test (OFT). The OFT was performed during daytime in a polyvinyl chloride plastic arena (1 × 1 × 1 m) illuminated with four 4.8 W (470 lm) light bulbs (Lexman, Leroy Merlin, Białystok, Poland) mounted above the corners (at 1.5 m height). Behavior was recorded remotely with a digital camera (Hero 5 GoPro Inc.) and controlled with a smartphone using the GoPro App (GoPro Inc.). Each animal was allowed to explore the arena freely for 5 min. Before each OTF, the arena was cleaned with 70% ethanol and dried.

Within 3 days of the OFT, the metabolic rate of each animal was measured using indirect calorimetry, as oxygen consumption in an open‐flow respirometry system. The outdoor air pumped to the system was dried (silica gel 2–5 mm, Chempur, Piekary Śląskie, Poland) and purified (activated coconut carbon 0.6–2.36 mm, Acrivsorb 109, Browin S.zoo, Łódź, Poland). During BMR measurements animals were kept within the thermoneutral zone (at ~30°C; Cygan 1985), in 300 mL custom‐made glass chambers placed in temperature‐controlled cabinets (model: KB 53, Binder GmbH, Tuttlingen, Germany). The flow through each chamber was set to ~250 mL·min^−1^ and measured incurrently (FlowBar‐8, Sable Systems International, Las Vegas USA; henceforth: SSI). A two‐line multiplexer (MUX, SSI) was used to sample air from two animals at a time and measure six individuals by sampling air automatically. The air leaving each chamber was sampled (at a rate of 100 mL·min^−1^) to pass through one of two gas analyzers (FC‐10a, SSI) connected to one of two multiplexer lines. Data were collected at 1 Hz using an analog–digital interface (UI‐2 SSI) connected to a PC running dedicated software (ExpeData software, SSI). During measurements, the air from each chamber was sampled for 2 min every 10 min. BMR measurements were conducted for 3–4 h, which allowed us to measure 12 individuals daily.

To induce and measure heterothermy, we performed continuous (sampled every few seconds), automatic *T*_b_ measurements during short‐term fasting (measurements lasted ~23 h; an additional hour was needed to switch animals for measurement) at moderate cold exposure (~16°C) within 2 weeks of capture (examples of *T*_b_ readings: Figure S1). Measurements were taken with the IPTT‐300 transponders and dedicated readers (FSP‐7005; Bio Medic Data Systems, Inc., Seaford, USA; henceforth: BMDS) connected to PCs by data acquisition stations (DAS‐8010; BMDS). Due to equipment limitations, only five individuals were measured daily. During measurements, animals were held in 850 mL chambers constructed from polypropylene containers (model: HPL 808, LocknLock Co., Seoul, Republic of Korea) and equipped with 4 g of wood shavings. Flow passing through each chamber was set at ~350 mL·min^−1^.

All physiological and behavioral traits were successfully measured in 113 individual mice (60 females and 53 males).

## 3 DATA ANALYSIS

### 3.1 Calculations

As a predictor of body condition, we calculated a scaled mass index (SMI) based on a standardized major axis regression between body mass (*m*_b_) and HW following Peig and Green (2009).

As a measure of exploration, we used the total distance moved during 5 min of the OFTs.

Oxygen consumption (V̇O_2_) was calculated following equation 10.2 of Lighton (2008) assuming a 0.8 respiratory exchange ratio. In total, at least 20 samples of V̇O_2_ were obtained for each individual during BMR measurements. Only the last 20 s of each sample were selected as the measure of individual metabolic rate. BMR was defined as the lowest metabolic rate value recorded for a given mouse.

Heterothermy was quantified for the 23 h *T*_b_ recordings using the heterothermy index (HI: Boyles et al., 2011). As raw intervals between readings of *T*_b_ varied from 3 s to 18 min, these data were averaged to always be the 20 min periods prior to the calculation of HI (examples in Figure S1).

Predation risk was assessed as the number of nights (yellow‐necked mice are strictly nocturnal: Wójcik & Wołk, 1985) on which any mouse predator was recorded (martens, foxes, badgers, and owls) over the subsequent five nights of camera trapping near the wooden boxes at each location.

### 3.2 Statistics

Data analysis was performed in R‐4.3.1.

The number of captured mice and the number of nights with predator presence were compared using a Mann–Whitney *U* Test.

Morphological, physiological, and behavioral variables were compared using generalized least squares (GLS) modeling with restricted maximum likelihood (REML) using the function ‘gls’ of the package ‘nlme’. In each GLS, the covariate dry total seed mass (TSM; the sum of seed mass collected at a given location) and the factors, i.e., sex and feeder presence were included. In the GLS for BMR, we included individual *m*_b_ as a covariate. We obtained a better fit between BMR and *m*_b_ using its log_10_‐transformed values. As heterothermy and behavior can be differently dependent on its components, we separated the overall *m*_b_ effect to body size and body condition when considering these traits. Thus, in the GLSs for heterothermy use and distance moved, we included the covariates HW and SMI. Model residuals for HI displayed heteroscedasticity resulting from the unequal variances for factors sex and feeder presence and, the unequal variance for the relationship between HI and TSM; hence, we used the ‘weights’ option of the function ‘gls’ to fix the constant variances (‘varIdent’) for factors and assume exponential variance structure (‘varExp’) for covariate. In each GLS, we tested the interaction between the feeder presence and the covariate TSM; however, we ultimately excluded it from the final models as it was insignificant. Moreover, we also checked if the factor location (included as a random effect in the linear mixed effect model using ‘lme’ function of ‘nlme’ package) significantly clusters the variance in studied traits, however, as it was found singular (see results: random locations were represented by single individuals), it was dropped from further analysis.

We used path modeling (PM) to make inferences on the plausible causal relationship between physiological and behavioral traits, as well as on the correlation between its variation and predation risk, natural food availability, *m*_b_, body size and body condition using the ‘piecewiseSEM’ package (Lefcheck, 2016). During PM, we carried out mixed effect modeling (LME) implemented in the function ‘lme’ of the R package ‘nlme’ to account for highly correlated predictors (feeder presence and predation risk: see results), with feeder presence included as a random effect (the first‐order autoregressive model was assumed for within‐subject structure) and predation risk as covariate. In the LME for HI, we accounted for unequal variance in the factor sex and the random effect feeder presence, as well as exponentially varying variance in the relationship between HI and TSM (see above). In each PM analysis, we included three LMEs: (1) log_10_‐transformed BMR adjusted for log_10_‐transformed *m*_b_; (2) HI adjusted for SMI, TSM, and sex (included as a binary variable); and (3) distance moved adjusted for TSM. The components included in each LME were selected as significant based on initial analysis for the given trait (see results: Tables 1 and 2). We ran a set of six path models assuming that the additional covariate number of nights with predator presence affected only one of the above traits, which then influenced anothers in all plausible scenarios (see results: Table 3). Both HI and distance moved were affected by the natural food resource availability (see results). We consequently tested whether this covariate sequentially state the traits (by building models assuming that natural food resources affected only one of the dependent variables). We used the corrected Akaike's Information Criterion (AICc – Burnham et al., 2011) to select the most informative among models of differing complexity. In total, we compared 18 path models, and the most explanatory one was tested with Fisher C statistics (the model was assumed to be significant when *p* > .05).

**TABLE 1:** Results of the generalized least square models explaining variation in morphological traits of female and male mice (Sex) captured at locations with (*n* = 13) and without (*n* = 18) supplementary food sources (Fedeer presence).

Model	Factors	*β* ± SE	|*t*‐Value|	*p*
Body size	Intercept	−0.59 ± 0.13	4.46	<.001
	**Sex (males)**	**1.40 ± 0.14**	**10.27**	**<.001**
	Feeder presence (feeder)	−0.11 ± 0.15	0.76	.452
	Natural food availability	0.01 ± 0.07	0.11	.916
	Natural food availability×Feeder presence	0.14 ± 0.14	1.00	.319
Body condition	Intercept	−0.36 ± 0.18	2.02	.046
	Sex (males)	0.20 ± 0.19	1.06	.290
	**Feeder presence (feeder)**	**0.41 ± 0.20**	**2.05**	**.043**
	Natural food availability	0.03 ± 0.10	0.29	.777
	Natural food availability×Feeder presence	0.55 ± 0.40	1.40	.166

*Note*: Body size – head width, Body condition – scaled mass index, Natural food availability – total dry mass of seeds. Bold‐statistically significant predictor.

**TABLE 2:** Results of the generalized least square models explaining variation in physiological and behavioral traits of male and female mice (Sex) captured at locations with (*N* = 13) and without (*N* = 18) supplementary food sources (Feeder presence).

Model	Factors	*β* ± SE	|*t*‐Value|	*p*
Maintenance	Intercept	−0.02 ± 0.10	0.25	.806
	**Body mass**	**0.92 ± 0.06**	**14.40**	**<.001**
	Sex (males)	−0.07 ± 0.13	0.57	.568
	Feeder presence (feeders)	0.09 ± 0.09	0.95	.344
	Natural food availability	0.00 ± 0.04	0.06	.949
	Natural food availability×Feeder presence	0.04 ± 0.09	0.39	.701
Heterothermy	Intercept	0.06 ± 0.15	0.40	.690
	Body size	0.03 ± 0.08	0.36	.717
	Body condition	−0.15 ± 0.08	1.80	.076
	**Sex (males)**	**−0.70 ± 0.15**	**4.75**	**<.001**
	**Feeder presence (feeders)**	**0.41 ± 0.16**	**2.50**	**.014**
	**Natural food availability**	**−0.15 ± 0.06**	**2.51**	**.014**
	Natural food availability×Feeder presence	−0.02 ± 0.14	0.13	.898
Exploration	Intercept	0.07 ± 0.18	0.38	.707
	Body size	0.04 ± 0.11	0.38	.706
	Body condition	0.11 ± 0.11	1.03	.303
	Sex (males)	0.10 ± 0.19	0.53	.600
	Feeder presence (feeders)	−0.18 ± 0.21	0.86	.393
	**Natural food availability**	**−0.23 ± 0.10**	**2.35**	**.021**
	Natural food availability×Feeder presence	0.09 ± 0.20	0.45	.651

*Note*: Maintenance – basal metabolic rate, Heterothermy – heterothermy index, Exploration – distance moved during open‐field test, Body size – head width, Body condition – scaled mass index, Natural food availability – total dry mass of seeds. Bold‐statistically significant predictor.

**TABLE 3:** List of the six most competitive path models explaining variation in maintenance metabolism under homeothermy, heterothermy, and exploratory behavior in yellow‐necked mice.

Model	AICc dsep	ΔAICc dsep	*k*
**Predators > Heterothermy(NFA) > Exploration(NFA) > Maintenance**	**87.70**	**0.00**	**23**
Predators > Heterothermy(NFA) > Exploration>Maintenance	94.18	6.48	22
Predators > Maintenance > Heterothermy > Exploration(NFA)	94.96	7.26	22
Predators > Heterothermy > Exploration(NFA) > Maintenance	98.30	10.60	22
Predators > Maintenance > Exploration(NF) > Heterothermy(NF)	98.94	11.24	23
Predators > Heterothermy(NFA) > Maintenance > Exploration(NFA)	99.58	11.88	23

*Note*: Predators – number of nights with predator presence, Heterothermy – heterothermy index, Exploration – distance moved during open‐field test, Maintenance – basal metabolic rate, NFA – Natural food availability as total dry mass of seeds (included or not as a covariate). Bold‐most explanatory path model.

## 4 RESULTS

In total, we collected 324.0 g of dry mass of acorns at all 44 locations (205.1 g at 31 locations where mice were captured) during the three autumn seed collection sessions. We also collected 239.1 g of dry mass of smaller seeds (146.4 g at locations where mice were captured), which were mainly hornbeam seeds (>95%). From the beginning of autumn to the moment of capture in winter, a total of 872.2 kg of wet mass of seeds (mainly hazelnuts ~80%) were left out at the 13 feeders.

The number of captured mice was higher at feeders (median = 6, range = 3–11; *N* = 13) than at control locations (median = 1, range = 0–5; *N* = 31, *U* = 7.00, *p* < .001). The number of nights with predators present was higher at feeders (median = 4, range = 1–5; *N* = 13) than at control locations (median = 0, range = 0–5; *N* = 31, *U* = 16.50, *p* < .001).

There was no interaction between natural food resources and feeder presence for morphological (Table 1), physiological, or behavioral traits (Table 2). Food resources did not affect body size, which was higher in males (mean ± SD = 16.13 ± 0.45 mm) than in females (mean ± SD = 15.38 ± 0.32 mm; Table 1). Body condition did not differ between sexes and was unaffected by natural food availability, but was slightly higher at locations with supplementary food sources (mean ± SD = 38.11 ± 4.78 g) than at control locations (mean ± SD = 37.11 ± 4.29 g; Table 1).

**FIGURE 2:**
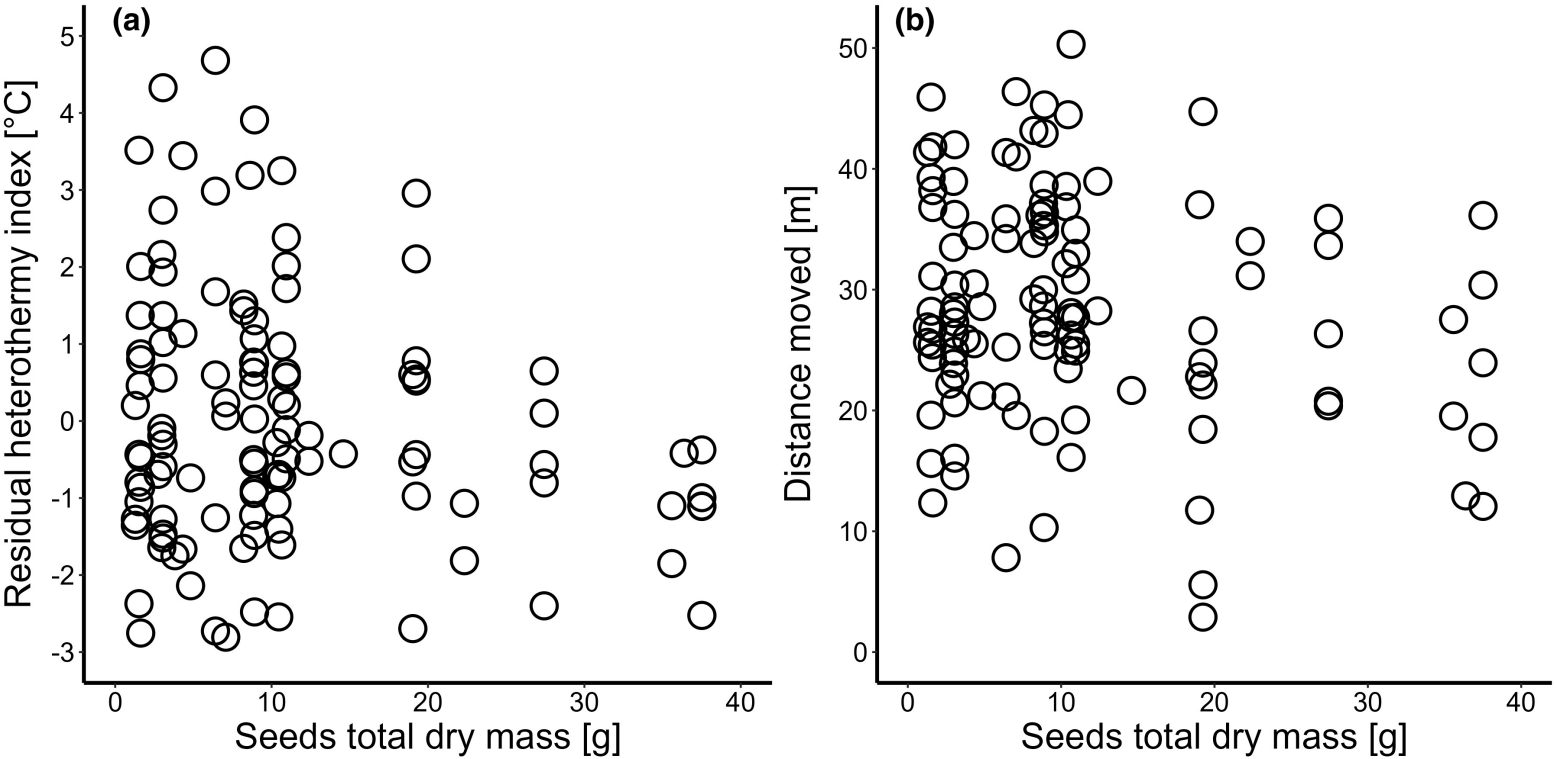
Relationships between the total dry mass of seeds collected at location and (a) the residual (sex and body condition adjusted) heterothermy index or (b) the distance moved in yellow‐necked mice.

Maintenance metabolism (basal metabolic rate—BMR) correlated positively with body mass (*m*_b_) and, *m*_b_‐adjusted BMR did not differ between the sexes and was affected neither by natural nor supplementary food sources (Table 2). Heterothermy (heterothermy index—HI) was not highly significantly affected by body size (head width—HW) and body condition (however, scaled mass index—SMI tended to be correlated negatively with HI; Table 2), but was higher in females (mean ± SD = 3.55 ± 1.74°C) than males (mean ± SD = 2.15 ± 1.56°C; Table 2). Sex‐adjusted HI correlated negatively with natural food availability (seed total dry mass; Figure 2a) but was also lower at control (mean ± SD = 2.44 ± 1.64°C) than at feeder locations (mean ± SD = 3.13 ± 1.83°C; Table 2). Body size, body condition, sex, and feeder presence did not affect exploration, and distance moved only correlated negatively with natural food availability (Table 2; Figure 2b).

**FIGURE 3:**
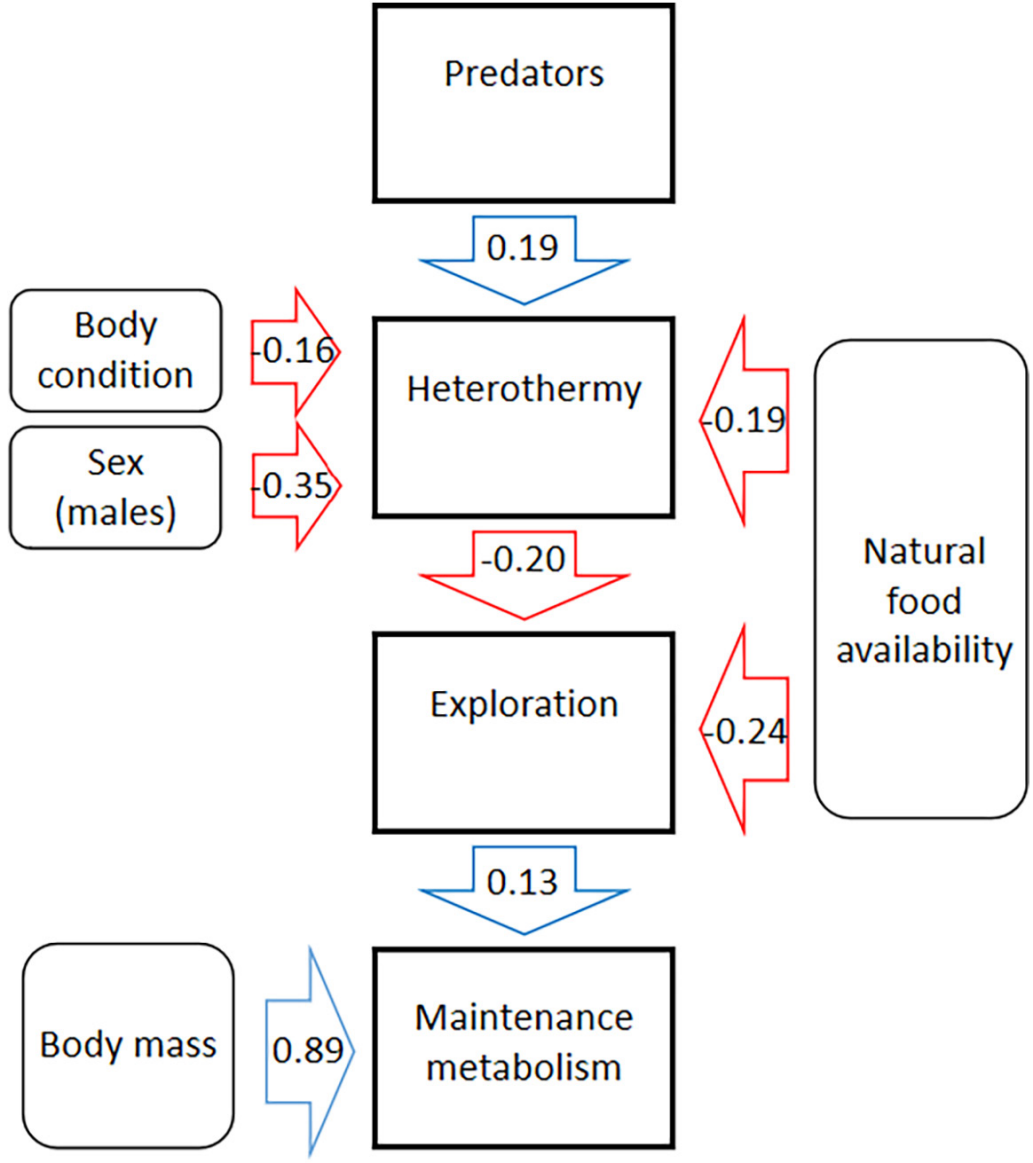
Most explanatory (see Table 3; Fisher *C* = 29.29, *p* = .082) path model describing causal relationships (arrows indicate causality) between behavioral and physiological traits, and morphological or ecological covariates as: Predators—number of nights with predator presence, Natural food availability—total dry mass of seeds, Body mass, Body condition—scaled mass index, Sex—binary variable (0—female, 1—male), Heterothermy—heterothermy index, Exploration—distance moved during the open—field test, and Maintenance metabolism—basal metabolic rate.

**FIGURE 4:**
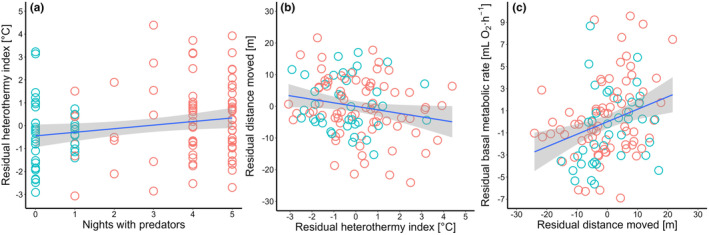
Relationships between (a) the residual heterothermy index (adjusted for sex, natural food availability, and body condition) and the number of nights with predator presence, (b) the residual distance moved (adjusted for natural food availability) and the residual heterothermy index, and (c) the residual basal metabolic rate (adjusted for body mass) and the residual distance moved. Green – random location, red – feeder location.

According to AICc, the best path model describing the correlations between behavioral and physiological traits assumed that predation risk first affects heterothermy use (Table 3; Figure 3; Tables S1, S2). According to this model, predation risk positively correlated with heterothermy use (Figure 4a; Table S2), which negatively affected exploration (Figure 4b; Table S2), which was then positively associated with maintenance metabolism (Figure 3; Figure 4c; Table S2).

## 5 DISCUSSION

The mice captured at feeders were in slightly better body condition, but supplementary feeding affected neither BMR nor exploration. Contrary to our predictions, feeder presence resulted in increased heterothermy use. The physiological explanation for this could be that the supplementary seeds were rich in polyunsaturated fatty acids, which have positive effects on torpor use (Ruf & Arnold, 2008). However, the overabundance of supplementary food and the correlated high density of mice at feeders were followed by increased predation risk. Mouse immigration (Stradiotto et al., 2009) and subsequently increased predation risk at locations where rodent abundance was high (Haapakoski et al., 2012; Jedrzejewski et al., 1995; Jędrzejewski & Jędrzejewska, 1996; Jedrzejewska & Jedrzejewski, 1998) could explain why we failed to find the predictable simple suppressive effect of supplementary food on use of energy‐saving thermoregulation.

The supplementary feeding did not result in expected differences in physiology and behavior, and at the same time, the feeder presence did not affect the relationship between natural food abundance and studied traits. Local abundance of natural food resources was negatively correlated with heterothermy (Figure 2a) and exploration (Figure 2b) but not with BMR (Table 2). The first correlation indicates that the use of an efficient energy‐saving strategy, such as torpor, is important from the perspective of the ‘food‐habits’ hypothesis and suggests that heterothermy can be regarded as a compensatory mechanism for high self‐maintenance costs under homeothermy (Boratyński et al., 2021; Cruz‐Neto & Bozinovic, 2004). Energy savings during torpor can allow an animal to allocate sufficient energy for self‐maintenance, even when living with high BMR—an evolutionary important trait associated with many aspects of animal performance that have fitness consequences (Arnold et al., 2021; Burton et al., 2011; Hulbert & Else, 2004; Sadowska et al., 2013; Speakman, 2005). For example, variation in maintenance metabolism can positively correlate with the rate of growth of individuals (Auer et al., 2020; Careau, Bergeron, et al., 2013; Sadowska et al., 2021), a trait crucial for survival in the natural environment. Similarly, our previous study found that maintenance metabolism was positively related to body mass gain in individual wild yellow‐necked mice born in masting and non‐masting years, despite their substantially different diets (Boratyński et al., 2021). However, individuals who experienced a scarcity of preferred food sources grew more slowly and went through winter with elevated resting metabolism and increased torpor use (Boratyński et al., 2021).

Direct examples of the effect of natural food availability on torpor use are scarce (review in Vuarin & Henry, 2014; see also: Pigeon et al., 2016; Siutz & Millesi, 2017; Hałat et al., 2020). Most of the field studies demonstrating any effect of resource availability on torpor use have been based on seasonal or among‐year comparisons. Studies that have measured thermoregulation in animals operating in natural conditions support the ‘torpor optimization’ hypothesis, which suggests the existence of a trade‐off between physiological and ecological costs, and the energy‐saving benefits of torpor use (reviews in Humphries, Thomas, et al., 2003, Humphries, Kramer, et al., 2003; Willis, 2017). This hypothesis predicts that individuals flexibly adjust torpor use in response to varying energy reserves and minimize heterothermy use (and associated ecological and physiological costs) when experiencing food abundance (Humphries, Thomas et al., 2003; Humphries, Kramer, et al., 2003; Landes et al., 2020). In this study, winter heterothermy in mice kept in the laboratory under food access *ad libitum* for about 2 weeks prior to measurements correlated with local food availability in autumn. We found in our previous study that heterothermy is highly repeatable in a studied population (individuals within‐population consistently differed in this trait, and 60%–90% of the total phenotypic variance in HI at the population level was explained by among‐individual differences; see Boratyński et al., 2019). The among‐individual phenotypic variation includes genetic component (Lynch & Walsh, 1998), and a level of repeatability sets upper limits for trait heritability (Dohm, 2002). However, the among‐individual differences can also be fixed during development, and the same genotype experiencing substantially different conditions may produce substantially different phenotypes (Piersma & Drent, 2003). From this perspective, our results imply that the ‘torpor optimization’ hypothesis can be applied to the results of microevolutionary processes and/or the permanent environmental effect. The negative correlation between heterothermy and natural food resources could have been a consequence of natural selection or developmental plasticity under food scarcity.

Torpor use has been hypothesized to be an emergency response to adverse conditions in individuals with poor body condition (Christian & Geiser, 2007). In our study, heterothermy tended to negatively correlate with body condition as assessed prior to measurements being carried out, supporting this assumption. However, many studies have shown also that individuals can use torpor when in good body condition (Stawski & Geiser, 2010; Vuarin et al., 2013; review in Nowack et al., 2017). This is likely because torpor has functions other than mitigating food scarcity, and one of those, among others, is predator avoidance (reviews in Geiser & Brigham, 2012; Nowack et al., 2017; Ruf & Bieber, 2023; Turbill et al., 2019; Turbill & Stojanovski, 2018; Vuarin & Henry, 2014). On the one hand, muscle performance is strongly temperature‐dependent (Bennett, 1984; James et al., 2015), and torpid animals are less responsive to stimulation (Speakman et al., 1991), which can result in them being more vulnerable to predator attack, at least when inactive in their roosts (our study species is predated in burrows by day‐active weasels, see Jędrzejewski et al., 1992). On the other hand, the reduced energy expenditure associated with this physiological state lowers the need for food acquisition and foraging, activities that may expose individuals to predation (Christe et al., 2006; Werner & Anholt, 1993). The higher number of nights with predator presence observed at locations with feeders suggests that access to increased food resources was associated with higher predation risk. Our path analysis indicates that increased predation risk at trapping locations was a primary predictor for increased heterothermy use (adjusted for variation in natural food resources) rather than for variation in mouse behavior or self‐maintenance of homeothermy. These results support the little‐studied predator–heterothermy interaction (reviewed in Nowack et al., 2017), suggesting that energy savings incurred during torpor allow animals to reduce their foraging time and thus, lower their exposure to predation, as predicted by comprehensive models of the ecological significance of adaptive hypothermia (Laurila et al., 2005; Pravosudov & Lucas, 2000). Only two experimental studies have provided (indirect) evidence that (presumed) increased predation risk (removed grass cover) results in increased torpor use by individuals (Turbill et al., 2019; Turbill & Stojanovski, 2018). This phenomenon requires more attention, especially in the context of testing the effects of surplus food availability on physiology in wild animals (Vuarin & Henry, 2014) where correlated increased predatory impact can be expected.

The path analysis indicated that if variations in energy‐saving strategy, self‐maintenance under homeothermy, and exploration are causally linked (besides predation risk primarily shaping torpor use), heterothermy (when also adjusted for the effects of natural food availability, sex, and body condition) negatively affected mouse exploratory behavior (adjusted for the effects of natural food resources), which in turn positively correlated with body mass‐adjusted BMR. The positive correlation between behavior and BMR is a pattern found across studies (reviewed in Mathot et al., 2019) and may support the ‘performance’ model rooted in the evolutionary ‘increased‐intake’ hypothesis (Careau et al., 2008; Careau & Garland, 2012). This model predicts that elevated BMR is required to improve the energy transformation process, which can then fuel proactive behavior and support the high daily energy expenditure resulting from costly activities. In turn, proactive behavior is expected to be responsible for increased energy gain via more efficient foraging (Careau & Garland, 2012; example in Wirowska et al., 2024). Interestingly, in our study, variation in behavior shaped variation in BMR rather than the opposite. Moreover, the maintenance costs of homeothermy were the last component of the path, suggesting that they are a consequence rather than a predictor of the other traits. Since torpor use can lower daily energy expenditure in natural conditions (Holloway & Geiser, 1995; Schmid & Speakman, 2000), heterothermic animals by employing an energy‐saving strategy are unconstrained/less constrained by the energy budget fixation or limitation assumptions of the ‘performance’ and the ‘compensation’ models. (Careau & Garland, 2012). The negative correlation between exploration and heterothermy suggests that animals using torpor do not need to be highly exploratory (after accounting for food abundance correlating with higher exploration: Figure 2b) since they already engage in a sufficiently energy‐saving strategy. We found heterothermy (as mentioned above), as well as BMR and exploration to be highly repeatable in our previous studies (Boratyński et al., 2019; Strijker et al., 2023); thus, the observed behavioral variation can be considered as animal personality (defined as consistent among‐individual differences in behavior across time and context: Réale et al., 2007). As the phenotypic correlation between repeatable traits indicates among‐individual correlation (review in Brommer & Class, 2017), our study presents the first empirical data suggesting that heterothermy (a strategy that can improve survival but impair reproduction; Dammhahn et al., 2017; Boratyński et al., 2024) is a significant component of a broader pace‐of‐life syndrome (Careau et al., 2008; Réale et al., 2010).

The population dynamics of the study species is strongly related to temporal synchronous seed production (masting) of the commonest forest tree species (Bogdziewicz et al., 2016; Pucek et al., 1993; Stenseth et al., 2002). During masting, predation risk experienced by individual mice is likely lowered due to the high mouse abundance at wide spatial distribution. Ongoing environmental changes are likely to substantially affect masting and dependent processes in forest systems (review in Medlyn et al., 2011), as warm winters, cold and heterogeneous springs combined with hot‐dry summers can lead to asynchronous phenology and flowering of main tree species (Bogdziewicz et al., 2017; Koenig et al., 2015). This may lead to local increases in food abundance (likely dependent on local water conditions—see Figure S2 for the correlation between elevation and seed production in our study), local mouse population growth, and subsequently increased predation pressure (reviewed in Jedrzejewska & Jedrzejewski, 1998). We suggest that in this context, torpor use could be a useful strategy for dealing with the indirect fallout of global climate change.

## AUTHOR CONTRIBUTIONS

**Jan S. Boratyński:** Conceptualization (lead); data curation (lead); formal analysis (lead); funding acquisition (lead); investigation (lead); methodology (lead); project administration (lead); resources (lead); validation (lead); visualization (lead); writing – original draft (lead). **Karolina Iwińska:** Investigation (equal); writing – review and editing (supporting). **Martyna Wirowska:** Formal analysis (equal); investigation (supporting); writing – review and editing (supporting). **Zbigniew Borowski:** Resources (supporting); writing – review and editing (supporting). **Karol Zub:** Funding acquisition (supporting); investigation (supporting); supervision (equal); validation (supporting); writing – review and editing (equal).

## CONFLICT OF INTEREST STATEMENT

No actual or potential conflicts of interest are declared by the authors.

## Supporting information


Appendix S1

Data S1

## Data Availability

All data are available in attached supplementary file (Data S1).
