# Supplementary material for: Predation can shape the cascade interplay between heterothermy, exploration and maintenance metabolism under high food availability
Source: Ecol Evol. 2024 Jun 25;14(6):e11579. doi: 10.1002/ece3.11579 (PMC11199196; doi:10.1002/ece3.11579)
Supplement: Supplementary file 1 — Appendix S1 [file ECE3-14-e11579-s001.docx]

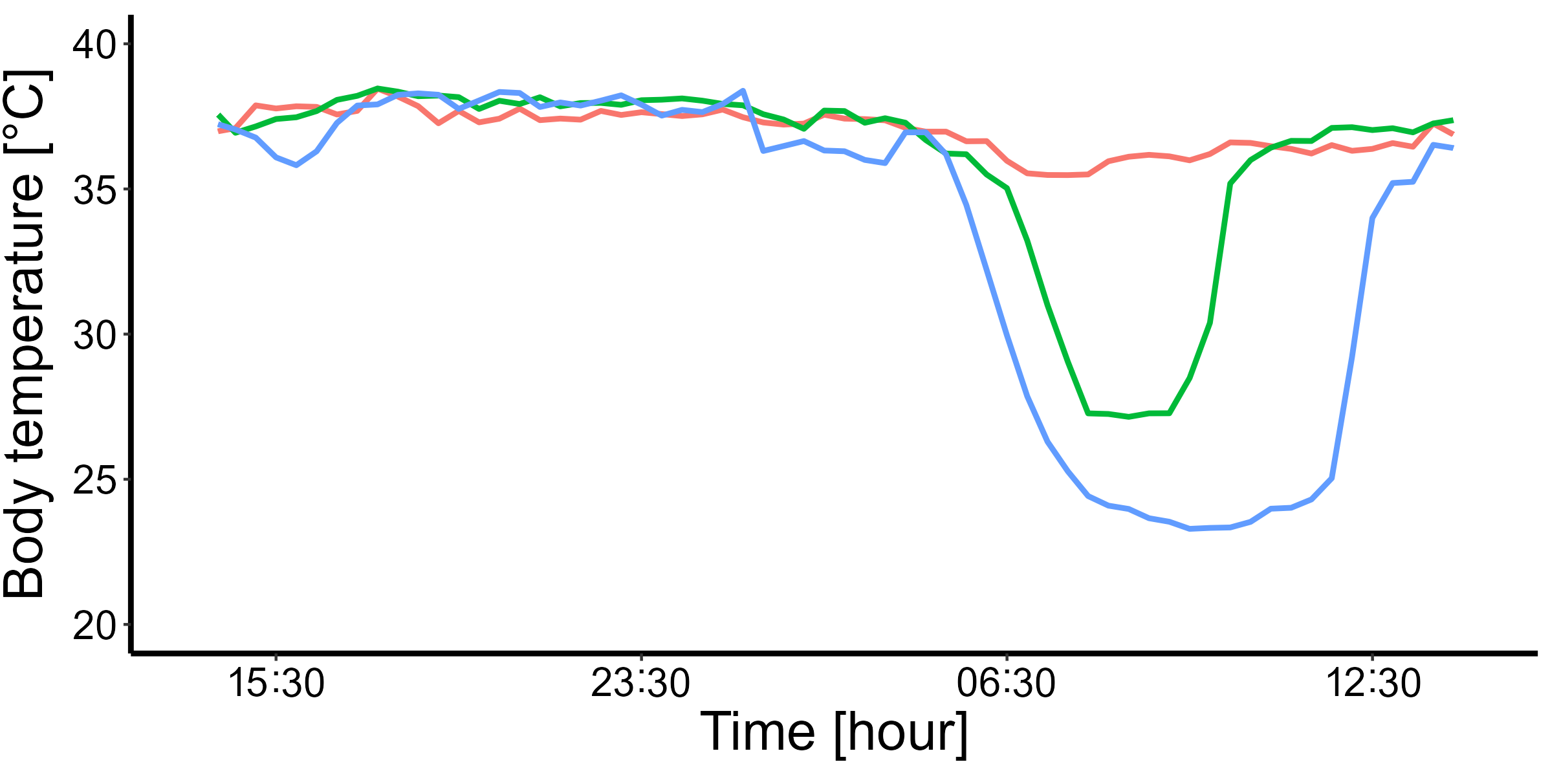


Figure S1. Time course of body temperature readings in three representative yellow-necked mice characterized by different heterothermy indices (HI - calculated following Boyles et al., 2011). Blue - HI = 7.1℃, green - HI = 4.0℃, red - HI = 0.9℃.


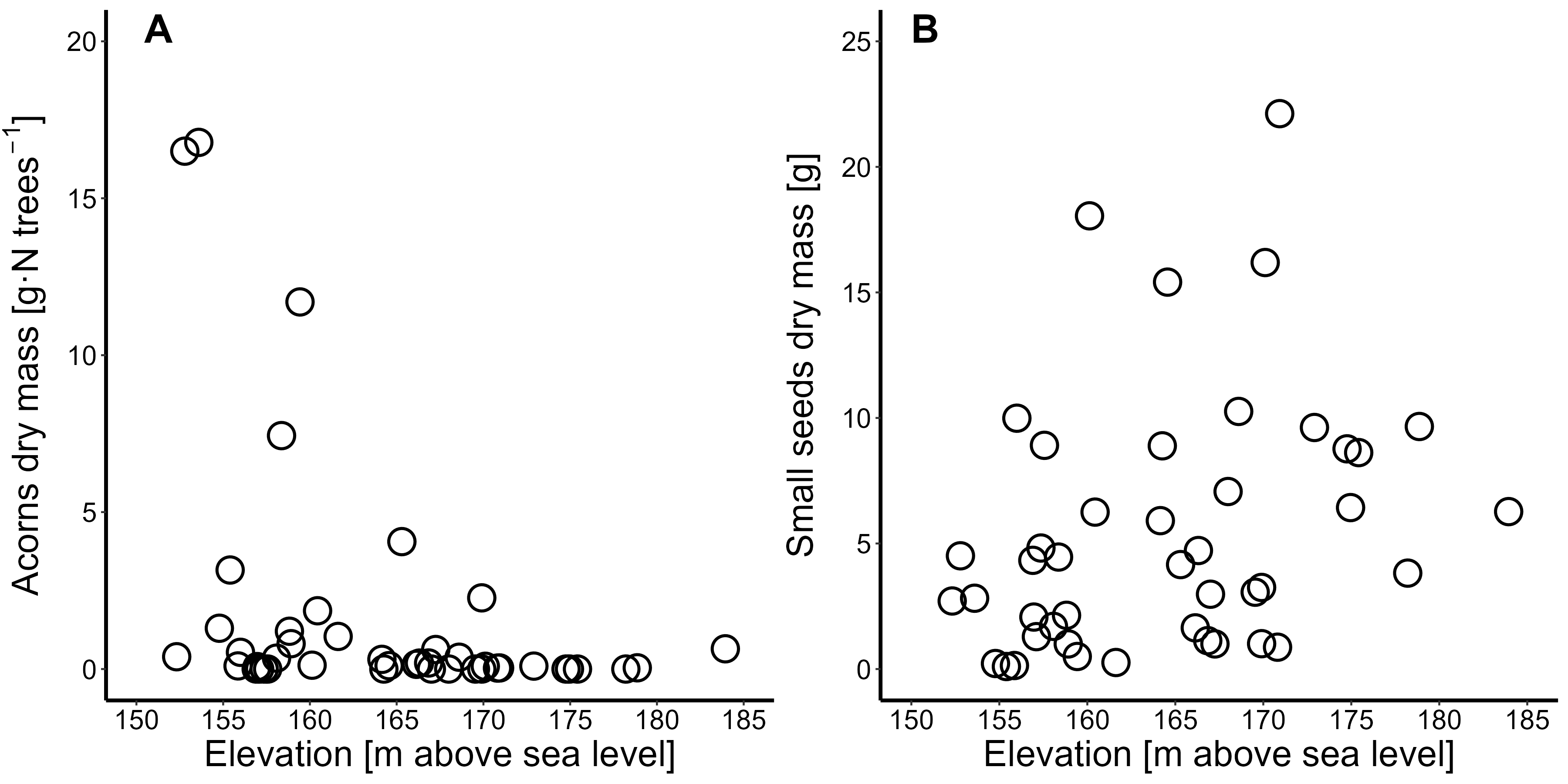


Figure S2. Relationship between elevation above sea level and A) average (per number of oaks) acorn dry mass and B) small seeds dry mass collected at given location during three subsequent sessions in autumn.

Table S1. Full set of competing path models explaining variation in maintenance metabolism, heterothermy and explorative behaviour in yellow-necked mice.

| Model | AICc dsep | ΔAICc dsep | k |
| --- | --- | --- | --- |
| **Predators>Heterothermy(NFA)>Exploration(NFA)>Maintenance** | **87.70** | **0.00** | **23** |
| Predators>Heterothermy(NFA)>Exploration>Maintenance | 94.18 | 6.48 | 22 |
| Predators>Maintenance>Heterothermy>Exploration(NFA) | 94.96 | 7.26 | 22 |
| Predators>Heterothermy>Exploration(NFA)>Maintenance | 98.30 | 10.60 | 22 |
| Predators>Maintenance>Exploration(NFA)>Heterothermy(NFA) | 98.94 | 11.24 | 23 |
| Predators>Heterothermy(NFA>Maintenance>Exploration(NFA) | 99.58 | 11.88 | 23 |
| Predators>Maintenance>Heterothermy(NFA)>Exploration(NFA) | 99.81 | 12.11 | 23 |
| Predators>Exploration(NFA)>Maintenance>Heterothermy | 99.93 | 12.23 | 22 |
| Predators>Maintenance>Exploration(NFA)>Heterothermy | 102.84 | 15.14 | 22 |
| Predators>Maintenance>Exploration>Heterothermy(NFA) | 103.42 | 15.72 | 22 |
| Predators>Heterothermy(NFA)>Maintenance>Exploration | 104.06 | 16.36 | 22 |
| Predators>Exploration(NFA)>Maintenance>Heterothermy(NFA) | 104.78 | 17.08 | 23 |
| Predators>Exploration(NFA)>Heterothermy(NFA)>Maintenance | 105.01 | 17.31 | 23 |
| Predators>Exploration(NFA)>Heterothermy>Maintenance | 106.62 | 18.92 | 22 |
| Predators>Maintenance>Heterothermy(NFA)>Exploration | 106.68 | 18.98 | 22 |
| Predators>Exploration>Heterothermy(NFA)>Maintenance | 106.98 | 19.28 | 22 |
| Predators>Exploration>Maintenance>Heterothermy(NFA) | 108.07 | 20.37 | 22 |
| Predators>Heterothermy>Maintenance>Exploration(NFA) | 108.77 | 21.07 | 22 |

Table S2. Table S1. Results of the most competitive path model explaining variation in maintenance metabolism under homeothermy, heterothermy and exploratory behaviour in yellow-necked mice. Predators – number of nights with predator presence, heterothermy – heterothermy index, exploration – distance moved during open-field test, maintenance – basal metabolic rate, condition - scaled mass index, food – natural food availability (included as a covariate), body mass and sex.

| Response | Predictor | Estimate | df | *P* | Standardized Estimate |
| --- | --- | --- | --- | --- | --- |
| Maintenance | Body mass | 0.65±0.03 | 109 | <0.001 | 0.87 |
| Maintenance | Exploration | 0.00±0.00 | 109 | 0.002 | 0.13 |
| Heterothermy | Condition | -0.05±0.03 | 107 | 0.038 | -0.16 |
| Heterothermy | Sex | -1.26±0.24 | 107 | <0.001 | -0.35 |
| Heterothermy | Food | -0.04±0.01 | 107 | <0.001 | -0.19 |
| Heterothermy | Predators | 0.17±0.06 | 107 | 0.010 | 0.19 |
| Exploration | Food | -0.24±0.09 | 109 | 0.010 | -0.24 |
| Exploration | Heterothermy | -1.07±0.49 | 109 | 0.032 | -0.20 |
